# Reduction of oxidative stress in total knee arthroplasty using tourniquet with a novel pharmaceutical combination

**DOI:** 10.1051/sicotj/2025042

**Published:** 2025-08-08

**Authors:** Irini Tatani, Electra Kalaitzopoulou, Marianna Skipitari, Aristeidis Ntoukas, Eleni A. Tsaliki, Savvas Giakoumakis, John Lakoumentas, Athina Varemmenou, Effimia Michail, Polyxeni Papadea, Christos D. Georgiou, Elias Panagiotopoulos

**Affiliations:** 1 Department of Orthopaedics, Patras University Hospital Patras 26504 Greece; 2 Department of Biology, University of Patras Patras 26504 Greece; 3 Department of Pharmacy, University of Patras Patras 26504 Greece; 4 Department of Medical Physics, School of Medicine, University of Patras Patras 26504 Greece; 5 School of Medicine, University of Patras Patras 26504 Greece

**Keywords:** Total knee arthroplasty, Tourniquet, Oxidative stress, Deferiprone, N-acetylcysteine, Postoperative pain

## Abstract

*Introduction*: Tourniquet use in total knee arthroplasty (TKA) can cause ischaemia-reperfusion (I-R) injury via oxidative stress. This study evaluated whether combined administration of the antioxidant N-acetylcysteine (NAC) and the iron-chelator Deferiprone can mitigate oxidative damage and improve clinical outcomes. *Materials and methods*: Twenty TKA patients were randomized into two groups, one group receiving NAC (600 mg, 6 h pre-op) and Deferiprone (1000 mg, 2 h pre-op) (intervention group) and the other group serving as placebo (control). Lipid hydroperoxides (LOOH) and protein malondialdehyde (PrMDA) were measured from quadriceps muscle tissue samples at 5 min (T1) and 40 min (T2) after tourniquet inflation, and 5 min after deflation (T3). Blood markers including serum ferritin, white blood cell (WBC) count, and polymorphonuclear neutrophils (PMNs) were assessed along with tissue PrMDA and LOOH as primary outcome measurements, while pain scores and knee flexion were recorded postoperatively as secondary outcome measurements. *Results*: LOOH levels were significantly lower in the intervention group at T2 and T3. PrMDA levels showed no significant differences. Ferritin levels rose by 69% in controls vs. 18% in the intervention group. WBC and PMNs normalized faster, with reduced pain and improved range of motion in the intervention group. *Conclusion*: The attenuation of LOOH elevation, the faster PMN deactivation, the inhibition of ferritin release from the cells along with the improved clinical outcomes suggest that combined NAC and Deferiprone administration may reduce tourniquet-related oxidative stress and inflammation, enhancing early recovery in TKA patients.

## Introduction

Tourniquets are commonly applied in elective total knee replacement surgery to reduce intraoperative blood loss and provide bloodless surgical exposure. However, tourniquet application induces ischemia in the lower limb, subsequently leading to anaerobic tissue metabolism, local lactic acidosis, and increased oxidative stress (OS) [1–3]. Once the tourniquet is released, reperfusion of oxygen-deprived tissues may augment the tissue damage produced by the ischemia alone. Clinically, I-R injury may present as increased postoperative pain, delayed wound healing, impaired muscle function, and, in some cases, remote organ dysfunction due to the systemic dissemination of proinflammatory mediators [3, 4]. Other complications of I-R injury include reduced protein synthesis, increased protein degradation, endothelial damage and upregulation of stress-pathway genes [3, 5].

The pathophysiology of I-R injury is multifactorial, with reactive oxygen species (ROS) production and leukocyte activation playing central roles [6]. OS arises when the generation of ROS – including superoxide radicals (O_2_•^−^), hydroxyl radicals (•OH), and hydrogen peroxide (H_2_O_2_) – exceeds the body’s antioxidant defence mechanisms [7, 8]. Among the key contributors to ROS-mediated damage is free iron (Fe), which catalyzes the Fenton and Haber-Weiss reactions, amplifying ROS generation and lipid peroxidation [9–12]. This cascade results in the accumulation of lipid hydroperoxides (LOOH) and malondialdehyde-protein adducts (PrMDA), both established biomarkers of oxidative tissue injury [7, 13, 14].

Ferritin, the primary intracellular iron storage protein, may also play a role in I-R injury. ROS can liberate iron from ferritin, further fuelling oxidative damage. Elevated serum ferritin levels have been correlated with OS and may serve as a marker of iron-mediated cellular injury [7, 10, 11].

Therapeutic strategies targeting these mechanisms include antioxidant administration and iron chelation. N-acetylcysteine (NAC), a precursor to glutathione (GSH), is an established antioxidant that replenishes intracellular GSH levels, a natural antioxidant, thus directly scavenging H_2_O_2_ and LOOH, which would otherwise react with free Fe^2+^ to generate ^•^OH radical [15]. Deferiprone is a clinically approved iron chelator that limits free iron availability, thereby reducing ROS generation via Fenton reaction. Both agents have been individually investigated for their cytoprotective effects in I-R models, though clinical evidence in the orthopaedic setting remains limited [16, 17].

Quantification of certain bi-products-markers of lipid peroxidation in biological systems is important to understand the role of ROS in OS-associated diseases. MDA (especially PrMDA) and LOOH are considered reliable markers of *in vivo* lipid peroxidation, and reflection of the molecular destructive magnitude of OS, while NAC has also been associated with reduced morphine consumption in RCTs, possibly implying a connection between OS and postoperative pain [14, 17]

The primary aim of this study was to assess whether the combined preoperative administration of NAC and Deferiprone can attenuate oxidative stress during TKA performed under tourniquet, as measured by blood inflammatory markers and LOOH and PrMDA levels in muscle tissue. The secondary aim was to evaluate whether this biochemical modulation translates into improved clinical outcomes, including reduced postoperative pain and enhanced recovery of knee function.

## Materials and methods

### Study design

The present study was a prospectively, placebo-controlled, single-centre trial, approved by the Scientific Board of Patras University Hospital. Eligible patients with advanced knee osteoarthritis scheduled for unilateral TKA between May 2017 and June 2019 participated in the study. All patients were fully informed about the purpose of this study and written informed consent was obtained from all participants according to the Helsinki Declaration. This study protocol was approved by the Institutional Ethical Committee (Protocol No. 200/04.05.2017).

Twenty female patients scheduled for unilateral TKA with the use of a pneumatic tourniquet were recruited. The inclusion criteria were age ≥ 55 years, capacity to understand information provided about the study, and signed informed consent. The exclusion criteria were dementia, haematological, metabolic, renal, and hepatic diseases, peripheral vascular disease, hypersensitivity or allergy to study drugs, contraindication to spinal anaesthesia and those already under treatment with corticosteroids or antioxidant agents.

All patients were randomly allocated by sealed envelopes to one of two groups: (a) 10 patients in intervention group P (Patient) T(Tourniquet)+N(NAC)+D(Deferiprone)+: those who received 600 mg of NAC (N; TREBON) per os 6 h before the operation (N+) and Deferiprone 1000 mg (D+; Ferriprox) per os 2 h before the operation at a dosage of 30 mg/Specific Serum Total Bilirubin (STB) and (b) 10 patients allocated in the control group P (T+N−D−) receiving placebo drugs per os 6 h and 2 h preoperatively. Regarding the baseline demographic characteristics of the two groups (BMI, age), no statistically significant differences were observed (Table 1).

**Table 1:** Baseline characteristics of patients.

Baseline data	P(T+N−D−), *n* = 10	P(T+N+D+), *n* = 10	*p*-value
Age, years	73 (71–75)	70 (69–73)	0.414
BMI, kg/m^2^	30.82 (26.85–33.06)	31.34 (28.12–33.75)	0.872

Data are shown as median (Q1–Q3). No statistically significant differences were noted between the two groups (*p* > 0.05; Wilcoxon’s rank-sum test).

The surgical team, participants, staff collecting data and performing assays, and statistician were blinded from the randomization.

### Perioperative care

All participants were admitted one day prior to surgery. All patients underwent a tricompartmental cemented TKA by a single surgeon using a medial subvastus approach and patellar retraction. At induction all patients were given intravenous Cefuroxime (1.5 g) which was continued for a further 24 h post-operatively, with two doses of 1 g. A tourniquet was applied to the proximal aspect of the thigh and inflated up to a pressure of 350 mmHg. All patients received subarachnoid anaesthesia by the same anaesthesiologist with 0.75% (7.5 mg/mL) ropivacaine (dose range 2–3 mL, depending on patient’s age and height). The same postoperative analgetic protocol was applied to all patients. During the first 48 h postoperatively, continuous epidural analgesia was provided with a constant infusion of 0.2% ropivacaine and 150 mcg clonidine at a total volume of 150 mL (1 mcg/mL), at a rate of 2 mL/h by catheter. The analgesia catheter was connected to a “patient-controlled analgesia” (PCA) pump, programmed to deliver a 0.5 mL dose with a 15-minute lockout interval. A wound drain was used and removed 48 h after the surgery. Patients also received low molecular weight heparin for prophylaxis against deep vein thrombosis, which was initiated on the first postoperative day.

### Specimen collection and outcome measurements

Blood and tissue markers were the primary outcome measurements assessed in this study. Blood samples for serum ferritin measurements were obtained before tourniquet inflation and after tourniquet release and analysed by radioimmunoassay. For complete blood count (CBC) analysis, blood samples were collected in typical ethylenediamine tetraacetic acid (EDTA) tubes and evaluated with an automated haematology analyser. CBC blood sampling was performed postoperatively on the day of surgery and daily thereafter until discharge. The white blood cells (WBCs) count and the percentage of polymorphonuclear neutrophils (PMNs) in the differential were recorded. Intraoperatively, quadriceps muscle tissue samples were obtained from the both groups of patients. The sampling was performed after 5 min of tourniquet inflation (Sample T1), after 40 min of tourniquet inflation (Sample T2), and 5 min after tourniquet deflation (Sample T3). Tissue samples were immediately frozen at −80 °C until further analysis, which included the quantification of two lipid peroxidation-derived OS-induced modifications: LOOH and PrMDA (their detailed protocol procedures are described in Supplementary material section). Postoperative clinical results were assessed as part of the secondary outcome measurements. During in-hospital stay, all patients were assessed for pain intensity using the visual analogue pain scale (VAS). Assessments for postoperative pain were performed on days 0 to 5, both at rest and during motion. The range of knee flexion was also recorded on days 0 to 5, using an orthopaedic goniometer.

### Statistical analysis

Quantitative variables collected includes preoperative and postoperative serum ferritin concentrations (ng/mL), oxidative stress markers [lipid hydroperoxides (LOOH) and protein-bound malondialdehyde (PrMDA)] at three intraoperative time points (T1, T2, T3), and postoperative VAS pain scores (at rest and during motion) recorded from postoperative day 0 to day 5. Knee range of motion (ROM), assessed daily and categorized as 0–30°, 30–60°, or 60–90°, represented an ordinal qualitative variable.

Data normality was assessed using the Shapiro-Wilk test. Due to the small sample size, all quantitative variables demonstrated non-normal distributions and were analyzed using non-parametric statistical methods.

To evaluate changes over time and intergroup differences:

- The relative change in serum ferritin (SF) was calculated as:

(1) $$\Delta SF\;=\;\frac{{SF}_{\mathrm{postop}}-{\mathrm{SF}}_{\mathrm{preop}}}{{\mathrm{SF}}_{\mathrm{preop}}}.$$

- Linear trend slopes were computed for each time-series variable (e.g., VAS, ROM, LOOH, PrMDA), using ordinary least squares (OLS) regression to quantify directional change. For analysis of ROM slopes, the ordinal scale was first converted to numeric values (1–3).
- All slopes were compared between groups and against a theoretical slope of 0 (indicating no change).

Group comparisons were conducted using:

- Wilcoxon rank-sum test for continuous or ordinal quantitative variables.
- Pearson’s chi-squared test for categorical variables.

All statistical tests were two-sided, with significance set at *p* < 0.05. Descriptive statistics are reported as median (interquartile range: Q1–Q3) for quantitative data and as absolute count (percentage) for categorical variables.

Data visualization included violin plots for group comparisons of distributional patterns. All statistical analyses and graphical outputs were performed using R (version 4.1.2, “Bird Hippie”) and RStudio (version 2021.09.1+372, “Ghost Orchid”).

## Results

### Laboratory findings

Regarding the SF, no significant intergroup differences were observed in absolute preoperative or postoperative serum ferritin values (Table 2). However, the perioperative variation in serum ferritin concentration (ΔSF) differed significantly between groups. The control group [T+N−D−] showed a median increase of 69%, compared to 18% in the intervention group [T+N+D+] (*p* = 0.029). The ΔSF change was also statistically significant within the control group (i.e., different from 0), but not within the intervention group.

**Table 2:** Serum Ferritin analysis of the groups. Serum Ferritin increased at a lower rate for the intervention group, indicating lower oxidative stress levels.

Group	T+N−D− patients, *n* = 10	T+N+D+ patients, *n* = 10	*p-*value (vs. group)
Preoperative SF (ng/mL)	62.5 (25.72–94.18)	82.2 (40.4–129.82)	0.436
Postoperative SF (ng/mL)	122.5 (49.95–132.75)	75.6 (39.88–161.5)	0.853
Variation of SF (Δc)	0.69 (0.26–1.12)	0.18 (0.01–0.42)	0.029*

Data are shown as median (Q1-Q3). Differences were analysed with Wilcoxon’s rank-sum test. *p < 0.05; statistically significant difference.

From CBC analysis, both groups exhibited an initial postoperative increase in WBC and PMNs on the day of surgery. However, normalization of WBC and PMN values occurred significantly earlier in the T+N+D+ group – often within the first 24 h – compared to the T+N−D− group (Table 3).

**Table 3:** Postoperative leukocyte normalization and polymorphonuclear neutrophil cell deactivation between the two groups.

Day of normalization	P(T+N−D−)	P(T+N−D−)	P(T+N+D+)	P(T+N+D+)
	Within day 1 post op	After day 1	Within day 1 post op	After day 1
WBCs < 11.5 K/L	40%	60%	90%	10%
PMNs < 80%	30%	70%	90%	10%

### Oxidative stress markers

**Figure 1:**
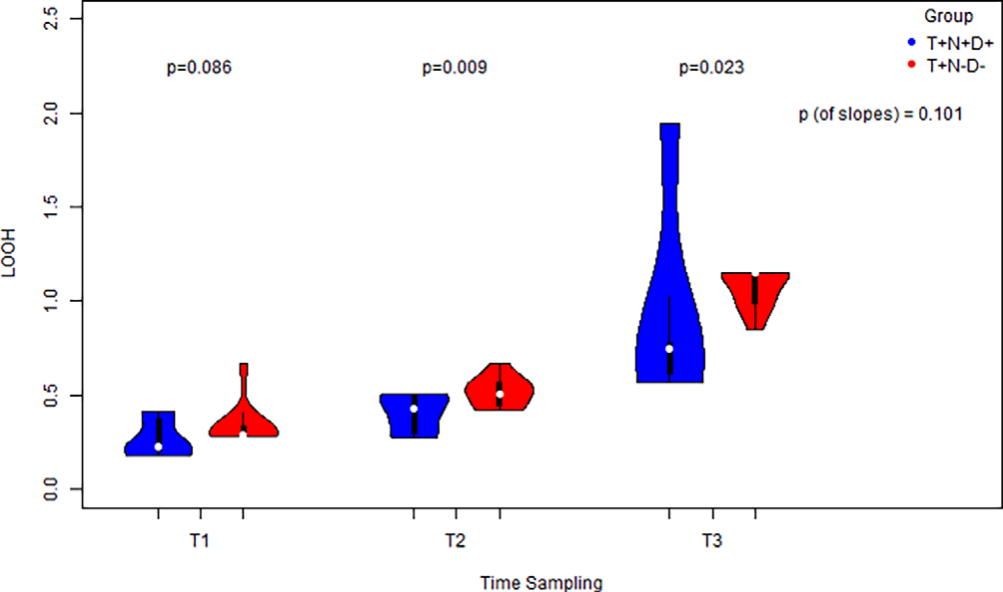
LOOH level measurement in cases vs controls at 3 distinct time points and a comparison for each distinct time point via the usage of side-by-side violin plots. Comparison of the equivalent slopes indicating an increase both for cases and controls.

LOOH concentrations increased in both groups over time. However, the control group exhibited significantly higher LOOH levels at time points T2 (40 min after tourniquet inflation) and T3 (5 min after deflation) compared to the intervention group (*p* < 0.05). No significant difference was observed at baseline (T1). Although the rate of LOOH increase (slope) over time did not differ between groups, the slope was significantly different from zero in both, indicating a true increase in oxidative stress (Figure 1).

**Figure 2:**
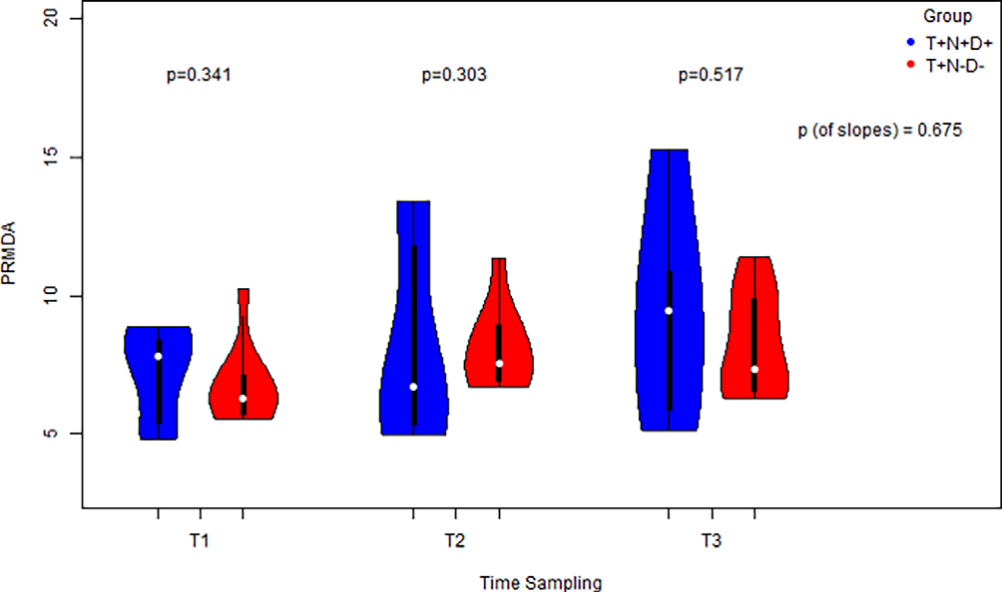
PrMDA level measurement in cases vs. controls at 3 distinct time points and a comparison for each distinct time point via the usage of side-by-side violin plots. Comparison of the equivalent slopes indicating non-monotonically an increase both for cases and controls.

PrMDA levels increased across all time points in both groups, but no significant differences were detected between groups at T1, T2, or T3 (Figure 2). Similar to LOOH, both groups showed significantly increasing slopes over time (slope ≠ 0), but the slopes did not significantly differ between the two groups.

### Clinical outcomes

**Figure 3:**
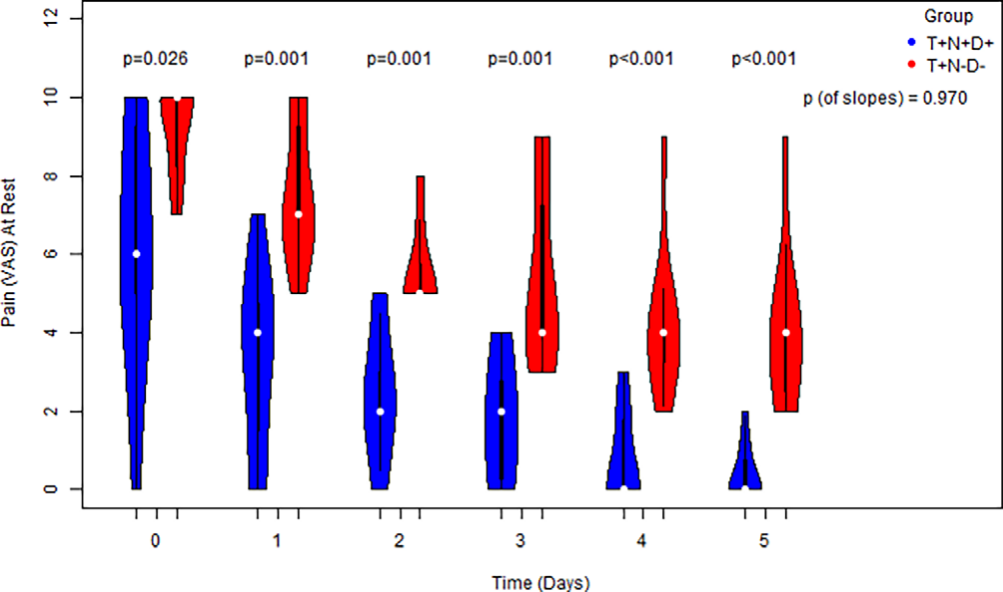
The progress of postoperative pain scores at rest of cases vs. controls for 0–5 days serially and a comparison for each distinct time point via the usage of side-by-side violin plots. Comparison of the equivalent slopes indicating a decrease in both groups.

**Figure 4:**
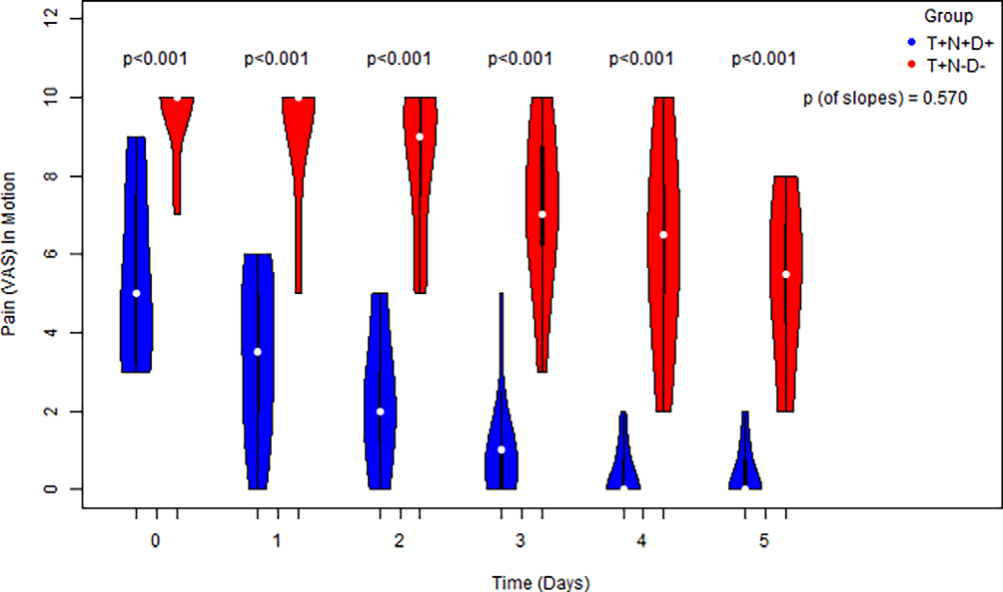
The progress of postoperative pain scores in motion of cases vs. controls for 0–5 days in sequence and a comparison for each distinct time point via the usage of side-by-side violin plots. Comparison of the equivalent slopes indicating a decrease in both groups.

Significantly lower pain scores were recorded in the T+N+D+ group compared to the control group on each of the five postoperative days, both at rest and during motion (*p* < 0.05 for each time point). While pain scores declined significantly over time in both groups (slope ≠ 0), the rate of pain reduction did not differ significantly between groups (Figures 3 and 4).

**Figure 5:**
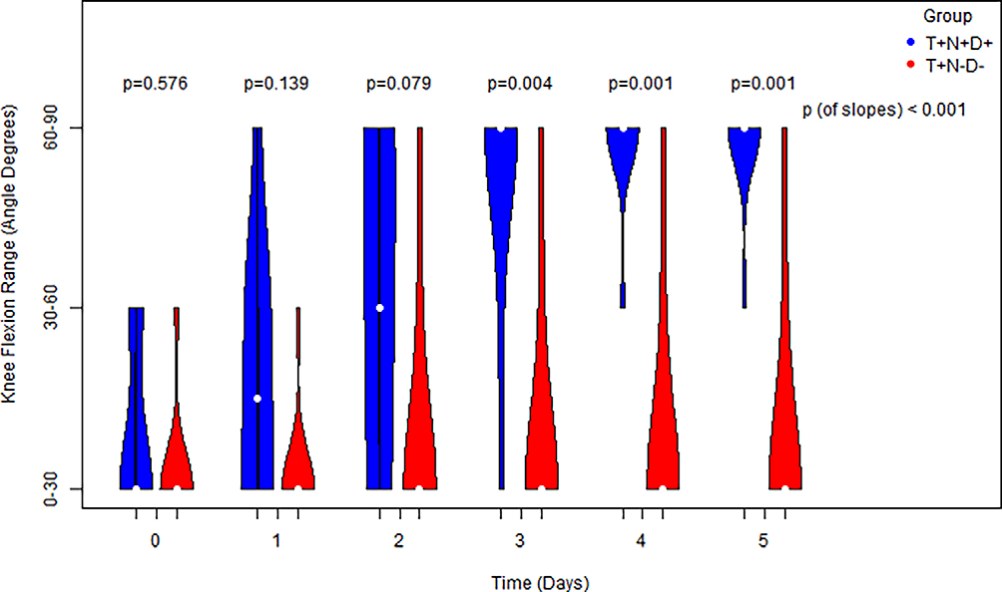
The progress of knee range of motion of cases vs. controls for 0–5 days serially and post-operatively and a comparison for each distinct time point via the usage of side-by-side violin plots, as well as comparison of the equivalent slopes, indicating an increase both for cases and controls.

Regarding the knee ROM, we found that there were no significant differences between groups on the day of surgery (*P* = 0.576), first day (*P* = 0.139), and second day (*P* = 0.079). However, T+N+D+ patients had significantly increased knee ROM compared to T+N−D− patients at postoperative days 3, 4 and 5 (*p* < 0.05) (Figure 5). The ROM improvement slope was significantly greater in the T+N+D+ group and statistically different from zero, suggesting continuous functional recovery. In contrast, the slope in the T+N−D− group did not differ from zero, indicating minimal postoperative improvement during the early recovery phase.

## Discussion

TKA with tourniquet application represents an excellent human model to study I-R induced tissue damage. Taking into consideration that Fe plays a fundamental role as a catalyst in the promotion and exacerbation of several OS induced disorders, we hypothesized that the combination of the antioxidant agent NAC with the free Fe chelator Deferiprone could be beneficial on reduction of OS and/or improvement of clinical outcomes in patients undergoing TKA with the use of a tourniquet [18, 19]. To the best of our knowledge, this is the first study to assess the antioxidant effects of the combined administration NAC with the free Fe chelator Deferiprone in routine TKA with the use of a tourniquet.

The strength of the present study is the simultaneous assessment of clinical outcomes and laboratory findings perioperatively. Although study findings are encouraging for the antioxidant effect of the combined use of NAC and free Fe chelator Deferiprone in patients undergoing TKA with the use of a tourniquet, we are aware of several limitations in this study. Limitations of this study include the small sample size and the short-term follow-up period and thus the results should be interpreted with caution. Nevertheless, our study results offer a first insight into the possible antioxidant effect of a novel combination of known pharmaceutical agents, which requires further evaluation to achieve maximal clinical effect.

Serum ferritin, a well-known Fe storage protein, has emerged as a biomarker not only in Fe-mediated diseases but also in OS related disorders. In this study we found that the mean variation in perioperative serum ferritin concentration was 69% in controls compared to 18% in the intervention group. Thus, it is obvious that co-administration of Deferiprone and NAC inhibits the release of ferritin from the cells. This finding allows us to speculate that the combination of these pharmaceutical agents may play a protective role against tissue damage and OS induced by the tourniquet application.

I-R causes cellular hypoxia in the affected tissues and subsequently leads to inflammatory responses that are followed by recruitment of white blood cells, especially polymorphonuclear cell type. Experimental models of I-R injury have identified the circulating polymorphonuclear leukocyte as playing a vital role in the pathogenesis of limb skeletal muscle injury [20]. In the present study, both WBC and percentage of PMNs were increased postoperatively on the day of surgery in both groups. Nevertheless, postoperative WBC and PMNs normalization occurred earlier in the T+N+D+ patient group. These results suggest that the combined administration of the NAC antioxidant and the Fe chelating factor Deferiprone may limit the amount of tissue damage induced by I-R and, therefore, may attenuate acute inflammatory response triggered by factors produced from the affected tissues.

The increase of lipid peroxidation in systemic circulation in patients undergoing knee surgery with tourniquet is well described in the literature [20, 21]. Lipid peroxidation induced after the production of ROS during ischaemia/reperfusion injury leads to oxidation of polyunsaturated fatty acids, disrupting membrane structure and producing degradation molecules such as LOOH and MDA, and its reaction product with proteins PrMDA [21]. In the present study, OS during TKA was assessed by measuring the marker of early-stage lipid peroxidation LOOH, and the late-stage lipid peroxidation marker PrMDA. LOOH levels were significantly increased after tourniquet release in the controls at T2 and T3 sampling points compared with the intervention group. In both groups, PrMDA levels were slightly increased after tourniquet release compared to the baseline measurement, but no statistically significant differences were noted between the two groups of patients at each sampling time. This may be due to PrMDA’s cumulative over time formation. Of note, PrMDA levels did not rise as expected after tourniquet release, as opposed to LOOH levels. This observation may be related to the fact that LOOH is an indicator of a short duration effect of OS, and as such, it is possibly developed during the early postoperative period, while PrMDA represents a long-lasting effect of OS.

Tourniquet use is common in TKA, but controversy exists regarding its use and effect on patient outcomes. Potential benefits include reduced intraoperative blood loss, better identification of anatomical structures, lower rate of transfusions, shorter surgical time, and increased accuracy in the positioning of the implant. Numerous disadvantages have been reported such as the elevated risk of perioperative complications, the increased pain intensity, and the slower functional recovery [20, 22]. However, its direct association with postoperative outcomes remains to be studied, as Bruehl et al. found in 2023 that increased OS is associated with long-term post-TKA pain outcomes but not with tourniquet use [23]. On the other hand, Grigoras et al. reported that tourniquet use in TKA is associated with lower blood loss, but similar postoperative pain and early functional outcomes compared to tourniquet-less surgery [24]. In the present study, patients in the intervention group showed significant improvement in pain scores both at rest and in motion as well as in functional result (range of knee flexion), compared to the control group. The improvement on clinical outcomes agreed with the corresponding reduction of OS markers (LOOH and SF), that noted in the intervention group.

Future studies could focus on the effectiveness of different methods of I-R injury attenuation such as ischemic preconditioning, vitamin C and propofol apart from the factors studied in this study [17, 19, 25]. Gender discrepancies in tourniquet usage are another field to be studied [24].

## Conclusion

This study confirms that TKA performed under tourniquet provides a reliable clinical model for investigating OS associated with I-R injury in the lower limb. The combined preoperative administration of NAC and the iron-chelator Deferiprone reduced ferritin release, accelerated normalization of postoperative WBC and PMN levels, and significantly attenuated LOOH elevation during early reperfusion, – markers all consistent with a mitigated oxidative response. These biochemical effects were accompanied by improved postoperative pain control and enhanced early functional recovery. Our findings suggest that this pharmacologic combination may offer a promising strategy for reducing tourniquet-related I-R injury in patients undergoing TKA.

## Data Availability

The original contributions presented in this study are included in the article/supplementary material. Further inquiries can be directed to the corresponding author(s).

## Supplementary material

The supplementary material to this article is available at https://sicotj.edpsciences.org/10.1051/sicotj/2025042/olm. Supplementary file supplied by the authors.
