# Supplementary material for: Reduction of oxidative stress in total knee arthroplasty using tourniquet with a novel pharmaceutical combination
Source: SICOT J. 2025 Aug 8;11:47. doi: 10.1051/sicotj/2025042 (PMC12334122; doi:10.1051/sicotj/2025042)
Supplement: Supplementary file 1 — Supplementary file supplied by the authors. [file sicotj-11-47-s1.pdf]

Supplementary Materials

Table of contents

S1. Materials 2
S1.1 Reagents 2
S1.2 Instrumentation 2
S1.3 Standard solutions 2
S2. Oxidative stress assessment protocols 3
S2.1. Sample tissue homogenization and fractionization procedure 3
S2.2. Protein concentration determination 4
S2.3. Oxidative stress (OS) assessment using LOOH and PrMDA assays 4

S1. Materials

S1.1 Reagents

Acetone, (iso)butanol, chloroform ($CHCl_3$), sodium hydroxide (NaOH), and methanol (MetOH) by Chem-Lab, ammonium iron (II) sulfate by Ferak, boric acid and sulfuric acid by Carlo Ebra, bovine serum albumin (BSA; fraction V) by Pan Biotech, butylated hydroxy anisole (BHA), deoxycholic acid (DOC; Na-salt), ethylenediaminetetraacetic acid (EDTA), ethanol (EtOH), hydrochloric acid (HCl), malonaldehyde bis (dimethyl acetal) 1,1,3,3-tetramethoxypropan (MDA), potassium chloride (KCl), sodium (di) hydrogen phosphate dihydrate ($NaH_2PO_4{\bullet}2H_2O$), sodium (tri) phosphate dodecahydrate ($Na_3PO_4{\bullet}12H_2O$), streptomycin sulfate (SS), 2-thiobarbituric acid (TBA), and trichloroacetic acid (TCA) by Sigma-Merck, Coomassie Brilliant Blue G250 (CBB), cumene hydroperoxide (Cum-OOH), and xylenol orange (XO; tetrasodium salt) by Alfa Aesar. All chemicals are of analytical grade, and their standard solutions are prepared from 18.2 mΩ ultra-pure water from a Milli-Q water system (Millipore Corp. Bedford, MA, USA).

S1.2 Instrumentation

- Balance (Kern, model 770/65/6J)
- Centrifugal vacuum concentrator (CHRIST, model RVC 2-18), connected to a vacuum pump (KNF, N 820.3 FT.18)
- Double-beam spectrophotometer (Shimadzu, model UV-1800)
- Homogenizer (Janke & Kunkel, model Ultra-Turrax T-25)
- Magnetic stirrer (FALC, model F30)
- Microcentrifuge (Thermo Scientific, model Pico 17)
- Microcentrifuge clear tubes, 1.5 and 2 ml (VWR, cat. no. 89000-028)
- Micropipettes 2.5 to 10, 20 to 200 µl, and 0.1 to 1 ml, adjustable volume (Eppendorf Research)
- Microcuvette (for absorbance measurements, 12.5x12.5x45 mm external dimensions, 4 mm internal window width, and 9 mm bottom thickness, 1.16 ml, quartz; Starna 9/B/9/Q/10)
- Microcuvette for fluorescence measurements (45x4 mm, 0.5 ml, quartz; Starna SOG/Q), fitted in a Starna, FCA 4 adapter
- pH meter (Metrohm, model 827 pH lab)
- Refrigerated microcentrifuge (HERMLE, model Z233 MK-2)
- Spectrofluorometer (Shimadzu, model RF-1501)
- Thermoblock (FALC, model TD-150-P1)
- Vortex (FALC, model MIX 10)
- Water bath (Memmert, model 920785)

S1.3 Standard solutions

- **0.66 M BHA**: Prepare 0.4 ml by dissolving 43 mg BHA in 0.360 ml absolute EtOH with vigorous vortexing for ~2 min.
- **Pi-EDTA-BHA homogenization buffer, pH 7.2**: Prepare 200 ml by dissolving 0.356 g Na_2HPO_4 and 0.074 g EDTA in ~195 ml ddH_2O, then add 0.3 ml 0.66 M BHA reagent, adjust pH dropwise with 1 M NaOH, and bring to final 200 ml with ddH_2O (final concentrations: 10 mM Na_2HPO_4, 1 mM EDTA, 1 mM BHA).
- **10% w/v SS**: Prepare fresh 1 ml by dissolving 0.1 g SS in ddH_2O to final 1 ml.
- **1 % w/v DOC**: Prepare 1 ml by dissolving 10 mg DOC in 1 ml ddH_2O.
- **100 % w/v TCA**: Prepare 10 ml by dissolving 10 g TCA in 3.8 ml ddH_2O.
- **$CHCl_3$:MetOH 2:1 v/v**: Prepare fresh by mixing two volumes $CHCl_3$ and one volume CH_3OH.
- **Cold acetone**: 100% acetone (~50 ml) is brought to -25°C.

- **50 mM borate buffer, pH 9**: Prepare 60 ml by dissolving 0.185 g boric acid in ~55 ml ddH_2O, adjust pH with 10 M NaOH, and bring to final 60 ml with ddH_2O.
- **0.74% w/v KCl**: Prepare1 ml by dissolving 7.4 mg KCl in 1 ml ddH_2O.
- **1 M and 50 mM NaOH**: The 10 ml 1 M NaOH solution is prepared by dissolving 0.4 g NaOH in 10 ml ddH_2O. The 20 ml 50 mM NaOH solution is prepared by 20x dilution of 1 M NaOH with ddH_2O (mixing 1 ml 1 M NaOH with 19 ml ddH_2O).
- **FOX reagent−Fe (2 mM xylenol orange, 0.25 M H_2SO_4)**: Prepare 10 ml by dissolving 7.6 mg xylenol orange, in 8 ml ddH_2O and addition of 140 µl H_2SO_4 (stock solution of 18 M). Stir the solution for 30 min (in RT, in the dark) until xylenol orange is dissolved, add ddH_2O final 10 ml and centrifuge at 10,000 *g* for 5 min at RT, to discard any undissolved xylenol orange remnants.
- **FOX reagent (+Fe)**: Prepare fresh 5 ml by dissolving 0.0076 g ammonium ferrous sulfate in 5 ml FOX reagent−Fe.

S2. Oxidative stress assessment protocols

S2.1. Sample tissue homogenization and fractionization procedure

Each of the ~0.5 g muscle tissue collected during operation time intervals is immediately frozen at -80°C until further treatment for the oxidative stress assessment, as follows:

Step 1. Weigh the muscle tissue (after removing extraneous blood with ddH_2O wash), mix with Pi-EDTA-BHA homogenization buffer at 1 ml for 1 g tissue sample proportion, and cool it in an ice-water bath. Homogenize the cold tissue (0.5 to 1 g was used) by an Ultra-Turax T25 homogenizer and centrifuge the homogenate at 10,000 *g* for 5 min at 4°C. Collect the supernatant and re-homogenize the pellet (down to its connective tissue remnants) with one volume cold homogenization buffer. Combine the two supernatants and measure their total volume.

Note: BHA in the homogenization buffer minimizes autooxidation of the tissue lipids, the fraction of which will be subsequently isolated to be analyzed for peroxidation.

Step 2. Add 0.111 ml SS stock solution for every one ml of the combined homogenate, incubate in an ice-water bath for 30 min, centrifuge at 10,000 *g* for 5 min at 4°C, and collect the supernatant (the pellet is discarded as it contains precipitated DNA and bound histone proteins). Then, add 20 µl 1% DOC per 1 ml of the resulting homogenate, incubate for 20 min at room temperature (RT), then add 0.12 ml 100% TCA per 1 ml of DOC-treated supernatant and incubate for 20 min in an ice-water bath.

Step 3. Phospholipids are isolated by a modification of the Folch procedure [1]. The DOC-TCA-treated supernatant is mixed (by vortexing for 30 sec) with an equal volume $CHCl_3$:MetOH 2:1, followed by centrifugation at 10,000 *g* for 5 min at 4°C. This, results in the formation of an upper aqueous, a middle disk (protein), and a bottom $CHCl_3$ (phospholipid) phase. The latter organic solvent fraction is carefully collected (by piercing the protein disk periphery with the narrow tip of a 200 µl-pipette), and the remaining two phases are 2x-washed with a volume of $CHCl_3$ equal to that of the collected organic fraction. The resulting three $CHCl_3$ volumes are combined, and vacuum-evaporated (in a speedvac CHRIST RVC 2-18 setup) to isolate the phospholipid (viscous liquid) pellet, which is washed with a mixture of 0.5 ml 0.74% w/v KCl and 0.5 ml $CHCl_3$:MetOH 2:1 by vortexing (for 30 sec), followed by centrifugation at 12,000 *g* for 5 min at 4°C. The resulting bottom $CHCl_3$ phase (phospholipids) is vacuum-evaporated to a viscous liquid down to a constant volume (40-150 µl), which can be stored -80°C for further analysis. If cloudiness is formed upon thawing, it is cleared by addition of ~10 µl MetOH.

Step 4. Subsequently, the disk-like protein from **step 3** is also collected (while the upper aqueous phase is discarded) and pelleted by washing with 0.5 ml 10% ice-cold TCA, followed by centrifugation at 10,000 *g* for 3 min at 4°C. The protein pellet is then 3x-washed with 0.5 ml

100% cold acetone by vortexing after smashing it into small portions by a glass rod, each time followed by centrifugation at 16,000 *g* for 5 min at 4°C. The protein pellet is vacuum-dried until it can be crumbled into a fine powder by a glass rod, and can be stored at -80°C.

Step 5. The resulting dried protein pellet is suspended in 1 ml 50 mM borate buffer (pH 9) to which are added 24 μl 1% DOC, and is incubated for 10 min at RT. Then, the protein is precipitated by the addition of 134 μl 100% TCA after incubation for 20 min in an ice-water bath, and centrifugation at 16,000 *g* for 5 min at 4°C. Subsequently, the protein pellet is 3x-washed with 0.5 ml 100% cold acetone (in order to remove any possible residues of DOC, TCA or hydrophobic contaminants), as described in **step 4**. At this step, the dried protein pellet can be stored at -80°C for further analysis.

Step 6. Protein pellets are solubilized in a minimum volume (~0.3 to 1 ml) 50 mM NaOH, by incremental addition of small volumes (50 μl) of this solvent and mixed by brief vortexing (and each time testing effectiveness of solubilization by centrifugation at 12,000 *g* for 1 min at RT).

S2.2. Protein concentration determination

Concentration of NaOH-solubilized protein pellet solution is determined on a small part of it (~10 μl) by a, previously reported, sensitive protein quantification assay [2], by assaying serial dilutions 100 to 1000-fold with ddH$_2$O.

S2.3. Oxidative stress (OS) assessment using LOOH and PrMDA assays

OS during TKA is measured by the indicators lipid hydroperoxides (LOOH), and malondialdehyde (MDA) protein (Pr) bound (PrMDA), expressed per mg sample protein, by modifications of their previously reported protocols [3] as follows:

LOOH assay: Small portions (1-50 μl) are drawn from the phospholipid liquid fraction (40-150 μl), obtained in **step 3** of sub-section ***S2.1***, are further diluted to 285 μl (with MetOH), and mixed, for sample (S) with 15 μl of FOX reagent+Fe, and for the sample blank (SB) with 15μl of FOX reagent−Fe. The assay also uses a reagent blank (RB) made by mixing 285 μl MetOH with 15 μl FOX+Fe (designated RB+Fe) and a reagent blank (RB) made by mixing 285 μl MetOH with 15 μl FOX−Fe (designated RB−Fe). Then, S, SB, RB+Fe and RB−Fe reaction mixtures are incubated for 30 min at RT in the dark, and their absorbance values are measured at 560 nm. Sample net absorbance is calculated by plotting the absorbance values in the following two straight line curves: One for the absorbance difference (S) − (RB+Fe), and the second for the absorbance difference (SB) − (RB−Fe), both versus the tested volume of the initial phospholipid liquid fraction (40-150 μl). The slope absorbance of the second curve is subtracted from the slope absorbance of the first, and this "net sample slope absorbance" is converted to LOOH (in CumOOH equivalents) using a CumOOH standard curve.

PrMDA assay**:** Small portions (~20-100 μl) from the solubilized (in 50 mM NaOH) protein pellets, obtained in **step 6** of sub-section ***S2.1***, are brought to 100 mM NaOH at final 250 μl (using a 1 M NaOH stock). The assay proceeds as described in the published protocols, and measurements are taken at ex/em 535/550 nm (setting the spectrofluorometer at 10 nm slit width, and at low sensitivity). The net FU sample value is determined by subtracting from the sample FU value the sample blank FU value and the reagent blank FU value, which is then converted to MDA concentration using MDA standard curve. For the particular samples used in the present study, the sample blank FU value was insignificant and was not accounted for.

References

1. Folch J, Lees M, Stanley GH. (1957). A simple method for the isolation and purification of total lipides from animal tissues. The Journal of biological chemistry, 226(1), 497–509

2. Georgiou, C. D., Grintzalis, K., Zervoudakis, G., & Papapostolou, I. (2008). Mechanism of Coomassie brilliant blue G-250 binding to proteins: a hydrophobic assay for nanogram quantities of proteins. Analytical and bioanalytical chemistry, 391(1), 391–403. https://doi.org/10.1007/s00216-008-1996-x

3. Grintzalis, K., Zisimopoulos, D., Grune, T., Weber, D., & Georgiou, C. D. (2013). Method for the simultaneous determination of free/protein malondialdehyde and lipid/protein hydroperoxides. Free radical biology & medicine, 59, 27–35. https://doi.org/10.1016/j.freeradbiomed.2012.09.038
